# Supplementary material for: Mid-infrared computational temporal ghost imaging
Source: Light Sci Appl. 2024 May 28;13:124. doi: 10.1038/s41377-024-01426-0 (PMC11133385; doi:10.1038/s41377-024-01426-0)
Supplement: Supplementary file 1 — Supplementary Information for mid-infrared computational temporal ghost imaging [file 41377_2024_1426_MOESM1_ESM.docx]

**Supplementary Information for**

**Mid-infrared computational temporal ghost imaging**

Han Wu,^1^ Bo Hu,^1^ Lu Chen, ^1^ Fei Peng,^2,*^ Zinan Wang,^3^ Goëry Genty,^4,*^ and Houkun Liang^1,*^

*^1 College of Electronics and Information Engineering, Sichuan University, Chengdu, Sichuan 610064, China^*

*^2 College of Electrical Engineering, Sichuan University, Chengdu, Sichuan 610064, China^*

*^3 Key Lab of Optical Fiber Sensing & Communications, University of Electronic Science & Technology of China, Chengdu, Sichuan 611731, China^*

*^4 Laboratory of Photonics, Tampere University, FI-33014 Tampere, Finland^*

*^Han Wu and Bo Hu contribute equally to this work.^*

Han Wu, Email: [hanwu@scu.edu.cn](mailto:hanwu@scu.edu.cn)

Bo Hu, Email: [hubo_uestc@hotmail.com](mailto:hubo_uestc@hotmail.com)

Lu Chen, Email: inkest@163.com

Fei Peng **(Corresponding author),** Email; pengfei@scu.edu.cn

Zinan Wang, Email: [znwang@uestc.edu.cn](mailto:znwang@uestc.edu.cn);

Goëry Genty **(Corresponding author)**, Email: goery.genty@tuni.fi;

Houkun Liang **(Corresponding author)**, Email: hkliang@scu.edu.cn

**Supplementary Note S1: Configurations and characteristics of the used pump sources in DFG process**

We have used two different types of fiber lasers as pump source for DFG depending on the target regime of TGI. Specifically, to perform the computational TGI in the 3.2-3.7 μm range, a tunable ytterbium-doped fiber laser from 1040 to 1090 nm is used as the pump source in DFG. To perform the computational TGI beyond 3.7 μm, a tunable random Raman fiber laser in the 1110-1150 nm range is used as pump laser in DFG.

The experimental layout of the tunable ytterbium-doped fiber laser in the 1040-1090 nm range is shown in **Figure S1a**. The laser consists of a tunable ytterbium-doped random fiber laser (YRFL) seed and power amplifier. A 976 nm laser diode (LD) is used as the pump source for YRFL seed, which is injected into a 6 m-long ytterbium-doped fiber (YDF, Nufern LMA-YDF-10/130) through a (2+1) × 1 combiner. A 1:1 coupler-based fiber loop mirror is connected to the signal port of the combiner and a wavelength tunable filter with 0.1 nm -3 dB bandwidth is integrated into the fiber loop mirror to provide a wavelength-selectable point feedback. Combining the active gain in the YDF, the wavelength-selectable point reflection, and the distributed random Rayleigh feedback in a 5 km-long single-mode fiber (SMF), a YRFL with tunable wavelength can be realized. The output power of the YRFL seed is 300 mW at 3 W LD pump. The YRFL seed is further boosted to 4 W in the power amplifier stage consisting of a 6 m-long YDF (Nufern LMA-YDF-10/130) and another LD pump. The tunable output spectrum is shown in **Figure S1b**. The tunable YRFL is then used as a pump for DFG with wavelength range from 3.2 to 3.7 μm.

The experimental design of the tunable random Raman fiber laser in 1110-1150 nm range is shown in **Figure S1c**. The laser uses the tunable YRFL as a pump. The tunable pump is injected into a 2 km-long SMF via the Pass port of a wavelength division multiplexer (WDM, Pass port: 1040~1090 nm, Reflection port: 1100~1700 nm). A 1:1 coupler-based fiber loop mirror is connected to the Reflection port of the WDM to provide broadband point feedback for random Raman lasing. The half-open cavity is therefore constructed by the fiber loop mirror and the random distributed Rayleigh scattering feedback in SMF. The output spectrum of the 1^st^-order random Raman fiber laser is shown in **Figure S1d**. It can be tuned from 1110 nm to 1150 nm with an output power up to 4W.


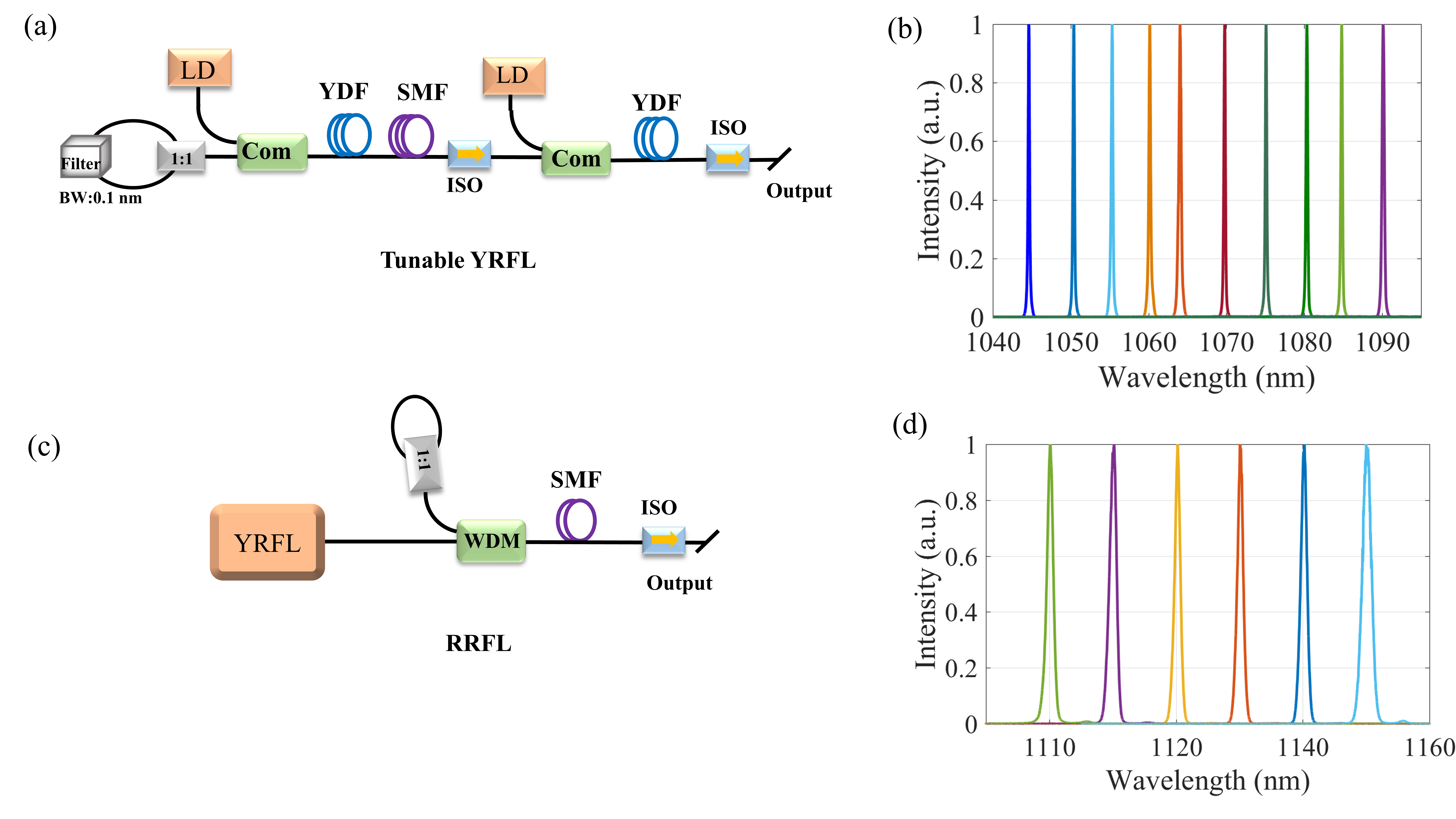


**Figure S1.** **(a)** Schematic of the YRFL design. WDM, wavelength division multiplexer; ISO, isolator; LD, laser diode; YDF, ytterbium-doped fiber; SMF, single mode fiber; Com, pump combiner. **(b)** YRFL output spectrum tunning from 1040 nm to 1090 nm. **(c)** Experimental layout of the RRFL. WDM, wavelength division multiplexer. **(d)** 1^st^ order RRFL output spectrum tunable from 1100 nm to 1150 nm.

**Supplementary Note S2: Preprogrammed patterns used in computational TGI and the source codes for data processing**

**Figure S2a** shows the generated 250 randomly chosen binary patterns uploaded to the AWG. **Figure S2b** shows the 32-order Hadamard matrix patterns where the −1 elements are substituted with 0. The source code to generate the 32-order Hadamard matrix patterns is also provided below.


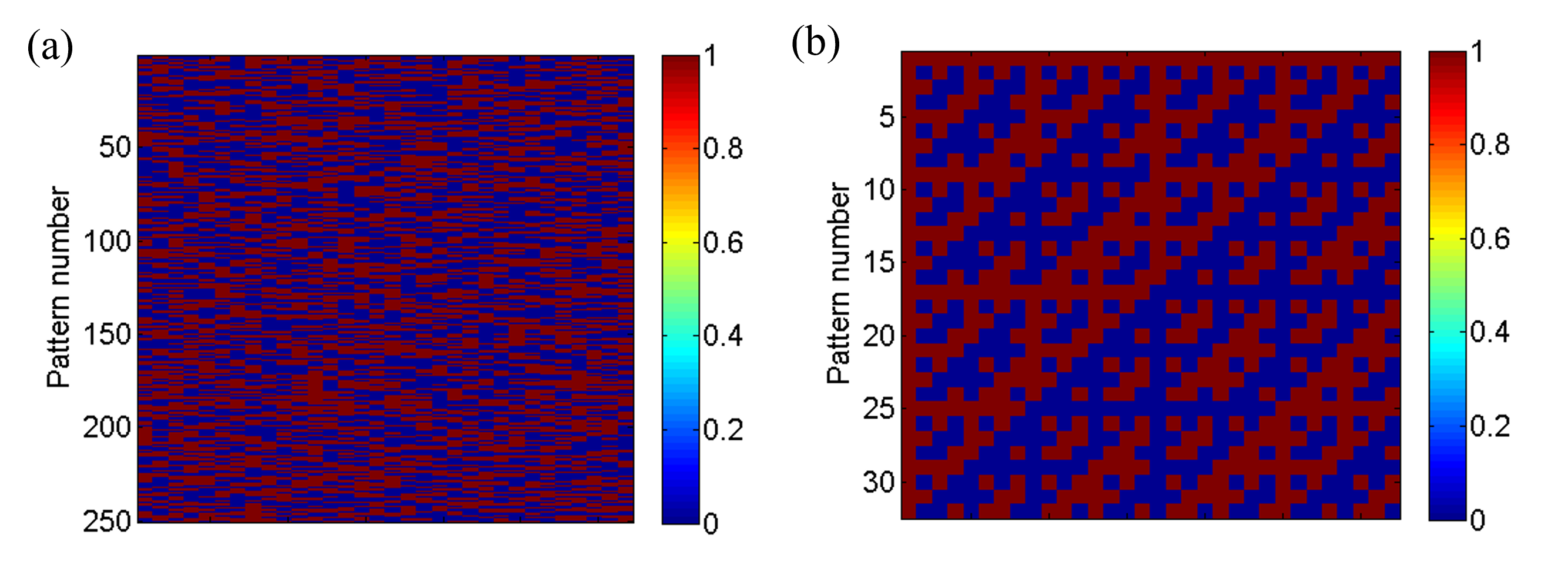


**Figure S2. (a)** The generated 250 realizations of randomly chosen binary patterns; **(b)** 32-order Hadamard matrix patterns

Source code for generating 32-order Hadamard matrix patterns where the −1 elements are substituted with 0:

caiY=1/10e6;

HadaPulse=ceil(1/10*10^-6/(caiY));

m=5;

Hada=[1];

for i=1:m

Hada=[Hada,Hada;Hada,-Hada];

i=i+1;

end

for i=1:size(Hada,1)

for j=1:size(Hada,2)

if Hada(i,j)==-1

Hada(i,j)=0;

end

end

end

for i=1:size(Hada,1)

H=repmat(Hada(i,:),HadaPulse,1);

HadaMatrix(i,:)=H(:)';

End

Source code for reconstructing the temporal object is provided below:

ref=load('aaa.Wfm.csv');%load the waveform of the preprogrammed patterns generated by AWG

buck=load('bbb.Wfm.csv');%load the waveform of the mid-infrared detector PDk (t)

n=250;%number of realizations

point=160;%number of sampling points in one realization;

ref=ref(1:point*n);

buck=buck(1:point*n);

buck1=reshape(buck,point,n);

int=sum(buck1,1);%calculate the time-integrated output of the mid-infrared detector B_k

ref1=reshape(ref,point,n);

obj=corr(ref1',int');% computing the second-order correlation between Rk (t) and Bk over n realizations

**Supplementary Note S3:** **Mid-infrared computational TGI results as a function of mid-infrared photodetector bandwidth**

In this section, to investigate the influence of bandwidth of mid-infrared photodetector on the quality of temporal object reconstruction based on the proposed computational TGI scheme, we performed the mid-infrared computational TGI for a 5 Mbps temporal object with 200 kHz, 500 kHz and 1MHz bandwidth of mid-infrared photodetector, respectively. The mid-infrared photodetector we used is a commercial HgCdTe (MCT) detector with adjustable gain selection (Thorlabs, PDAVJ5). The effective bandwidth of this MCT detector changes with the different gain settings. **Figure S3** shows the effect of the gain switch setting on the respective output frequency response of the used PDAVJ5 mid-infrared detector. We also experimentally measured mid-infrared pulsed light with different pulse durations by using the PDAVJ5 detector at different gain settings. From the datasheet and the experimental measurement, we can estimate the effective bandwidths of the PDAVJ5 mid-infrared detector are 200 kHz, 500 kHz and 1MHz at gain setting 8, 7 and 4, respectively.

**
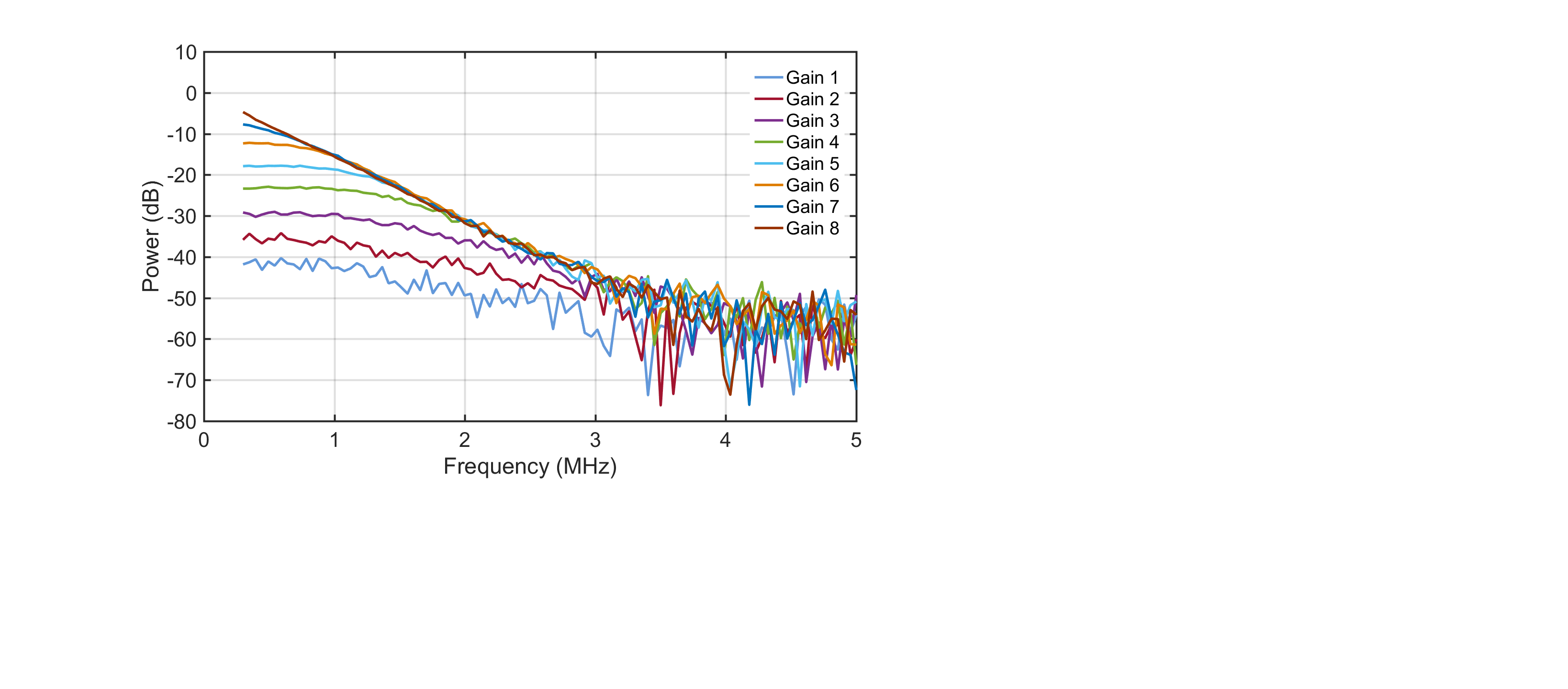
**

**Figure S3.** The effect of the gain switch setting on the respective output frequency response of the used PDAVJ5 mid-infrared detector.

In the mid-infrared computational TGI, we detect the mid-infrared light after interacting with the temporal object by using the PDAVJ5 mid-infrared detector at gain setting 8, 7 and 4, respectively and then reconstruct the ghost imaging of the 5 Mbps temporal object with 200 kHz, 500 kHz and 1MHz bandwidth of mid-infrared photodetector, respectively. The example recorded waveforms (PD*_k_*(*t*)) of the mid-infrared light after interacting with the temporal object which are detected by the mid-infrared photodetector at 1 MHz, 500 kHz and 200 kHz bandwidth are shown in **Figure S4**. In our TGI signal recovery algorithm, we use time-integrated output of the mid-infrared detector as the bucket signal, so that the $B_{ko}=\int_{0}^{T} {PD}_{k}(t')dt'$, the temporal object is reconstructed by computing the second-order correlation between *H_k_* (*t*) and *B_k_* calculated over the 32 realizations. **Figure S5** depicts the comparison of TGI results for a 5 Mbps temporal object with 200 kHz, 500 kHz and 1 MHz bandwidth of mid-infrared photodetector, and the results confirm the mid-infrared computational TGI works successfully at all 3 detector bandwidths.


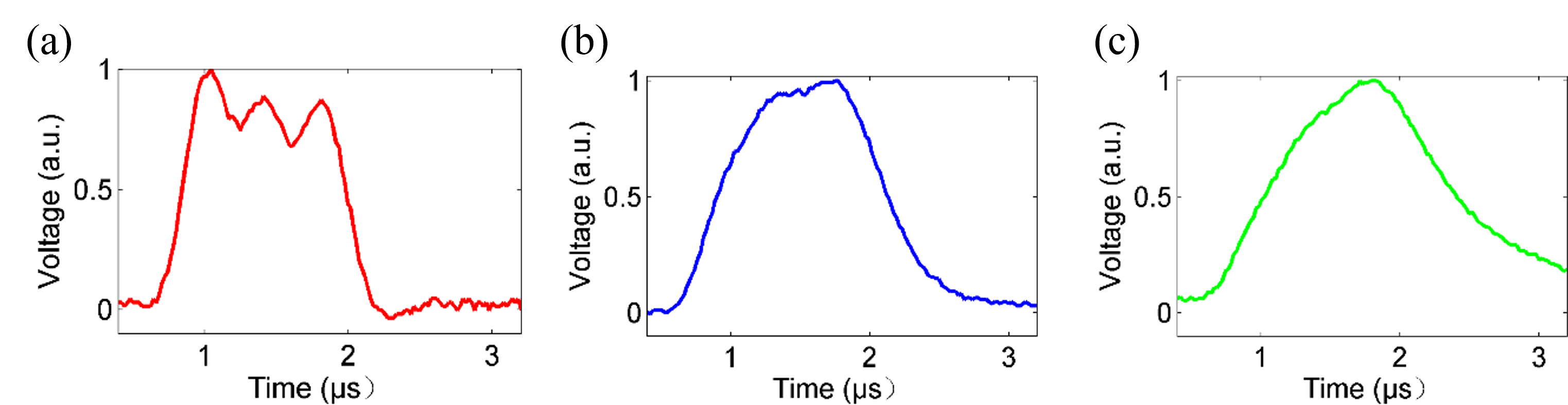


**Figure S4.** The example recorded waveforms (PD*_k_*(*t*)) of the mid-infrared light after interacting with the temporal object which are detected by the mid-infrared photodetector at (a)1 MHz, (b) 500 kHz and (c) 200 kHz bandwidth.


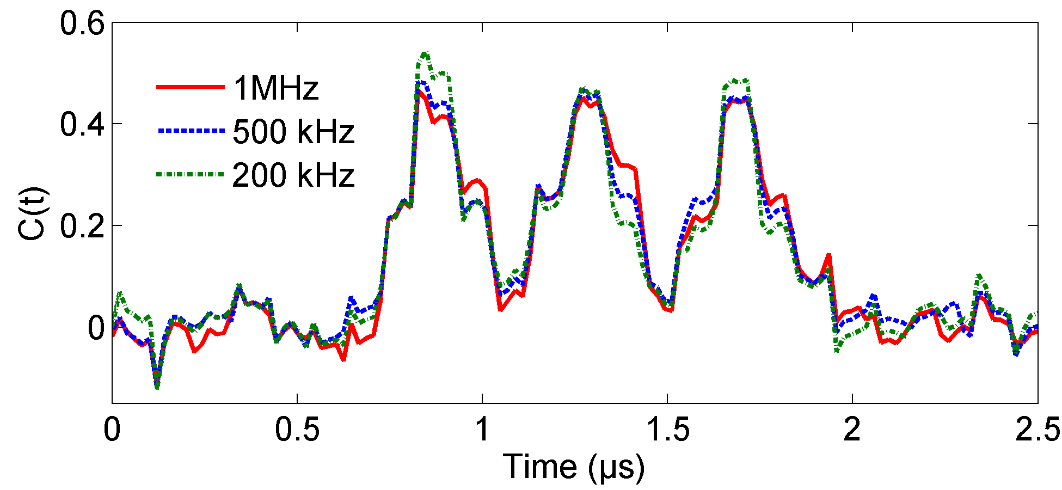


**Figure S5.** The comparison of TGI results for a 5 Mbps temporal object with 200 kHz, 500 kHz and 1 MHz bandwidth of mid-infrared photodetector.

**Supplementary Note S4: Side-by-side comparison with existing TGI methods**

In this section, we compare the requirements on laser sources, detectors or modulators to perform TGI in mid-infrared region and the TGI performance in terms of temporal resolution and number of measurement realizations based on different TGI methods, as shown in **Table S1**. The TGI methods for comparison include the two-arm detection TGI proposed in Ref. S1, two-color detection TGI proposed in Ref. S2, direct implementation of computational TGI proposed in Ref. S3, and the frequency downconversion based computational TGI proposed in this work.

**Table S1** Side-by-side comparison with existing TGI methods to operate in mid-infrared region

| **TGI methods** | **Laser source** | **Modulator** | **Detector** | **Temporal resolution** | **Number of realizations** |
| --- | --- | --- | --- | --- | --- |
| **Two-arm detection TGI^S1^** | Mid-infrared laser with random temporal fluctuations | / | ***Fast mid-infrared detector*** and slow mid-infrared detector | Determined by fluctuation time of the light source and the bandwidth of fast mid-infrared detector | Very large |
| **Two-color detection TGI^S2^** | Mid-infrared laser with random temporal fluctuations | / | Fast near-infrared detector and slow mid-infrared detector | Determined by fluctuation time of the light source and the bandwidth of fast near-infrared detector | Very large |
| **Direct computational TGI^S3^** | Mid-infrared laser | ***Fast mid-infrared intensity modulator*** | Slow mid-infrared detector | Determined by modulation bandwidth of mid-infrared intensity modulator | Small |
| **Frequency downconversion based computational TGI** | Near-infrared lasers | Fast near-infrared intensity modulator | Slow mid-infrared detector | Determined by modulation bandwidth of near-infrared intensity modulator | Small |

From the **Table S1,** we can conclude that due to the lack of commercially available fast detector and intensity modulator in mid-infrared region, the two-arm detection TGI and direct computational TGI cannot be easily applied in mid-infrared region with good temporal resolution performance. On the other hand, two-color detection TGI requires a mid-infrared laser source with random intensity fluctuations as well as a large number of measurement realizations. Frequency downconversion based computational TGI proposed in this work can not only relax the requirements on the laser sources, detectors or modulators in mid-infrared region, but also enable high performance TGI in terms of temporal resolution and number of realizations due to the use of ultrafast near-infrared intensity modulator and the preprogrammed orthogonal temporal patterns.

**Supplementary Note S5: Application of frequency downconversion based computational TGI in mid-infrared free-space optical communications**
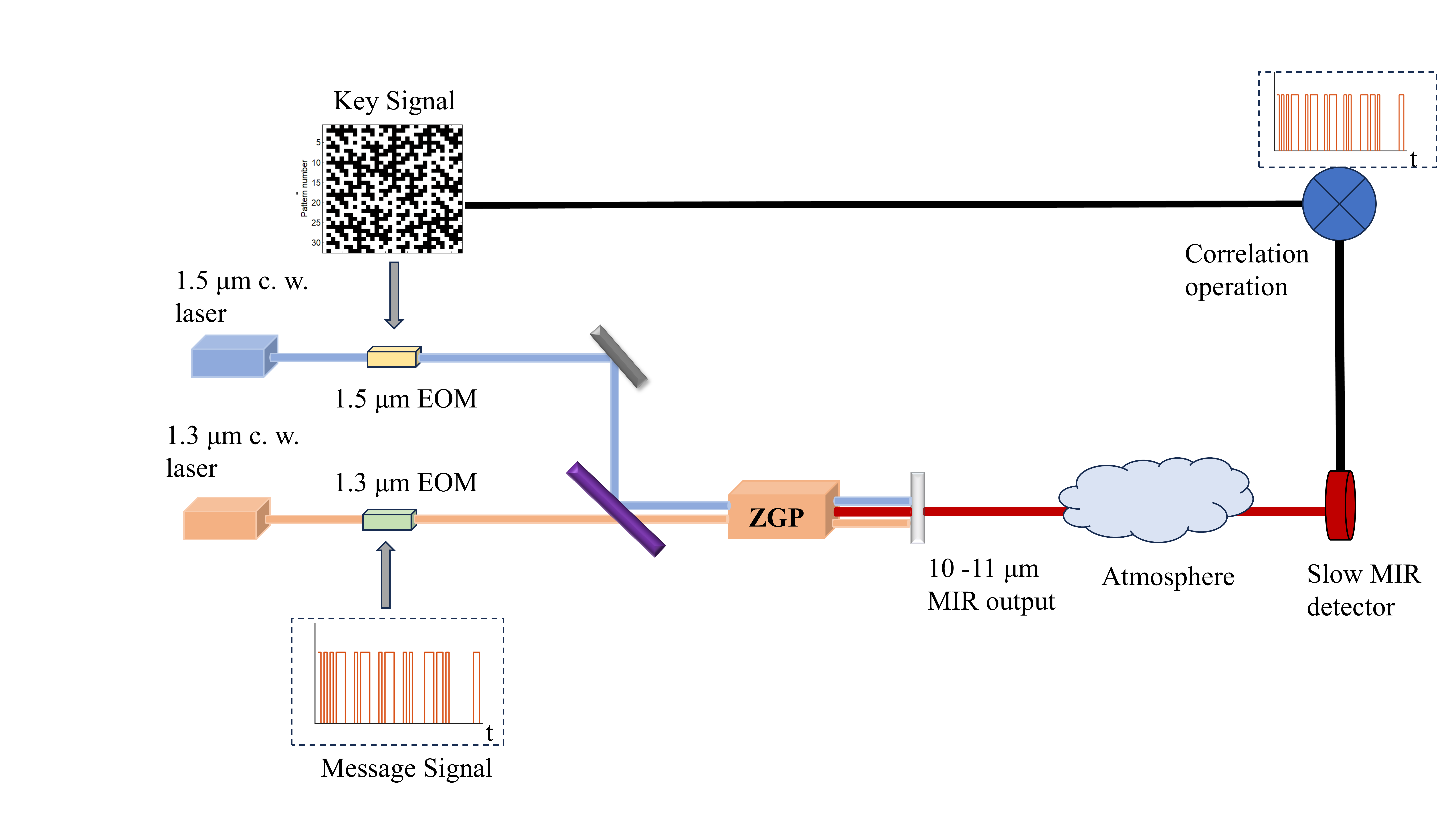


**Figure S6.** A possible schematic of frequency downconversion computational TGI based reliable high-frequency signal transmission and secure communication in the mid-infrared region.

A possible schematic of frequency downconversion computational TGI based reliable high-frequency signal transmission and secure communication in the mid-infrared region is shown in **Figure S6.** The preprogrammed temporal patterns (such as randomized Hadamard matrix patterns for secure communication) modulate the transmission of 1.5 μm EOM and therefore the temporal intensity of the 1.5 μm light. The “message” signal to be transmitted modulate the transmission of 1.3 μm EOM and therefore the temporal intensity of the 1.3 μm light. Though DFG in ZGP crystal, idler light located in 10-11 μm mid-infrared region generated with the temporal intensity $I_{idler}(t)\propto I_{1.5\mu m}(t)\times I_{1.3\mu m}(t)$. Therefore, for the mid-infrared light, the “message” signal is optically encrypted by a sequence of randomized orthogonal secret key. After free-space transmission, the mid-infrared light would be detected by the slow mid-infrared detector, and the intensity integrated over a particular bit sequence is $B_{k}=\int_{0}^{T} {I_{1.5 \mu m}}_{k}(t')I_{1.3 \mu m}(t')dt'$. The “message” signal can be reconstructed by computing the second-order correlation between randomized orthogonal secret key and *B_k_* over the *N* distinct bit sequences.

**SUPPLEMENTARY REFERENCES**

[S1] P. Ryczkowski, M. Barbier, A. T. Friberg, J. M. Dudley, and G. Genty, “Ghost imaging in the time domain,” *Nat. Photonics* **10**, 167–170 (2016).

[S2] H. Wu, P. Ryczkowski, A. T. Friberg, J. M. Dudley, and G. Genty, “Temporal ghost imaging using wavelength conversion and two-color detection,” *Optica* 6, 902–906 (2019).

[S3] Y. K. Xu, S. H. Sun, W. T. Liu, G. Z. Tang, J.Y. Liu, and P. X. Chen, “Detecting fast signals beyond bandwidth of detectors based on computational temporal ghost imaging,” *Opt. Express* **26**, 99-107 (2018).
